# MET Activation by a Macrocyclic Peptide Agonist that Couples to Biological Responses Differently from HGF in a Context-Dependent Manner

**DOI:** 10.3390/ijms19103141

**Published:** 2018-10-12

**Authors:** Wenyu Miao, Katsuya Sakai, Ryu Imamura, Kenichiro Ito, Hiroaki Suga, Tetsushi Sakuma, Takashi Yamamoto, Kunio Matsumoto

**Affiliations:** 1 Division of Tumor Dynamics and Regulation, Cancer Research Institute, Kanazawa University, Kakuma, Kanazawa 920-1192, Japan; miaowy1988@gmail.com (W.M.); k_sakai@staff.kanazawa-u.ac.jp (K.S.); imamura@staff.kanazawa-u.ac.jp (R.I.); 2 Department of Chemistry, Graduate School of Science, The University of Tokyo, Hongo, Bunkyo-ku, Tokyo 113-0033, Japan; fobishness1025@gmail.com (K.I.); hsuga@chem.s.u-tokyo.ac.jp (H.S.); 3 Department of Mathematical and Life Sciences, Graduate School of Science, Hiroshima University, 1-3-1 Kagamiyama, Higashi-Hiroshima, Hiroshima 739-8526, Japan; tetsushi-sakuma@hiroshima-u.ac.jp (T.S.); tybig@hiroshima-u.ac.jp (T.Y.); 4 Nano Life Science Institute, Kanazawa University, Kakuma, Kanazawa 920-1192, Japan; 5 Tumor Microenvironment Research Unit, Institute for Frontier Science Initiative, Kanazawa University, Kanazawa 920-1192, Japan

**Keywords:** HGF, MET receptor, surrogate agonist, partial activation, signal transduction

## Abstract

Non-native ligands for growth factor receptors with distinct chemical properties and different biological activities have the potential to become therapeutic applications. We previously generated MET/hepatocyte growth factor (HGF) receptor agonists using bivalent macrocyclic peptides. The highest MET-activating agonists exhibited biological activity that was indistinguishable from the effects of HGF. In this study, we investigated MET activation, signal characteristics, and biological responses induced by a macrocyclic peptide partial agonist known as aML5-PEG11. aML5-PEG11 induced weak tyrosine phosphorylation of MET while enhancing cell migration with potency comparable to HGF. aML5-PEG11 induced marked AKT (protein kinase B) and ERK (extracellular signal-regulated kinase) activation at a comparable potency and time-dependency to HGF, which suggests that enhancement of cell motility is attributable to activation of these molecules. In a 3-D culture of bile duct cancer cells in collagen gel, HGF induced robust activation of MET, ERK, and AKT, which was associated with enhanced expression of genes involved in bile duct development and subsequent branching of tubulogenesis. In contrast, aML5-PEG11 induced marginal activation of MET, ERK, and AKT (levels near the detection limits), which was associated with failure to enhance the expression of genes involved in bile duct development and a lack of tubulogenic response. Thus, MET activation by aML5-PEG11 couples to biological responses differently from HGF in an extracellular context-dependent manner.

## 1. Introduction

Growth factors and cytokines (collectively referred to as growth factors in this paper) exert biological activities by binding to and activating their membrane-spanning receptors. Ligand-induced homo-typic or hetero-typic dimerization of the receptors triggers activation of intracellular signaling pathways, which leads to altered gene expression profiles and biological responses. Engineered ligands and natural ligands have the capability to exert partial or modulated downstream signaling by influencing the stability of the receptor-ligand complex, affinity, and/or dimer conformations [1,2,3,4,5,6,7]. Attempts to generate non-native growth factor receptor agonists with distinct chemical properties and different capabilities to activate receptors have been conducted by using different approaches including variant growth factors with deletion and/or amino acid replacements, synthetic compounds, oligonucleotides, and peptides [8,9,10,11,12].

The activation of the MET receptor by its natural ligand known as the hepatocyte growth factor (HGF) triggers various cellular responses including cell proliferation, cell scattering/migration, and epithelial branching morphogenesis in a 3-D matrix [13,14,15]. These biological activities mediated by the MET receptor pathway supports embryogenesis and tissue regeneration [13,14,15] where each biological activity contributes differently to the biological processes in a cell type-related manner or tissue type-related manner. Design and generation of MET-agonistic molecules indistinguishable from native ligands and partially agonistic molecules with selective biological activities have the potential to be applicable to medical use.

Recently, we generated several bivalently linked macrocyclic peptides with the capacity to dimerize and activate the MET receptor by using the random nonstandard peptides integrated discovery (RaPID) system [12]. The bivalent macrocyclic peptides showed full-agonism or partial agonism toward the MET receptor depending on the linker length for the macrocyclic peptides. The fully MET-agonistic macrocyclic peptides induced intracellular signal activation profiles with a time profile and potency similar to HGF. Furthermore, the fully MET-agonistic macrocycles induced epithelial tubulogenesis and up-regulated a set of functionally classified genes involved in multicellular organism development in a manner that was indistinguishable from HGF.

In this study, we identified the bivalent macrocyclic peptide aML5-PEG11 as a partial MET-agonist. aML5-PEG11 partially activated the MET receptor and coupled to activation of downstream signal molecules and biological responses differently from HGF in an extracellular context-regulated manner. We elucidated the underlying mechanism leading to the different biological responses between aML5-PEG11 and HGF from the aspect of MET receptor activation, activation of intracellular signal transducers, and gene expression.

## 2. Results

### 2.1. MET Receptor Activation and Biological Responses

**Figure 1:**
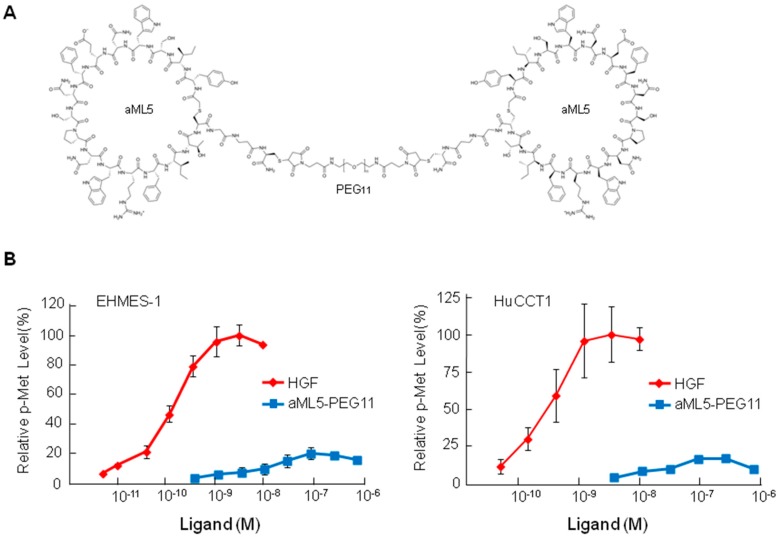
Chemical structure of aML5-PEG11 and MET receptor activation by aML5-PEG11. (**A**) Structure of aML5-PEG11. (**B**) MET receptor activation by aML5-PEG11. EHMES-1 and HuCCT1 cells were treated with aML5-PEG11 or HGF for 10 minutes. MET receptor phosphorylation at Tyr 1234/1235 was quantified by using a cell-based phospho-MET receptor assay. Each value indicates the mean ± SD obtained from independent experiments performed in triplicate.

aML5-PEG11 is composed of two aML5 MET-binding macrocyclic peptides linked by polyethylene glycol (PEG) (Figure 1A). To evaluate the potency of aML5-PEG11 toward the MET receptor, Y1234/1235 phosphorylation of the MET receptor was detected by using a cell-based ELISA in cultured EHMES-1 human mesothelioma cells (Figure 1B, left). HGF induced Y1234/1235 phosphorylation of the MET receptor in a dose-dependent manner and achieved maximal phosphorylation at 1–10 nM. Maximal aML5-PEG11-induced MET Y1234/1235 phosphorylation occurred at 100 nM. However, the maximal efficacy of aML5-PEG11 was about 20% of that observed with HGF. We confirmed the reduced efficacy of aML5-PEG11 to induce Y1234/1235 phosphorylation in another cell line known as the HuCCT1 human bile duct cancer cells (Figure 1B, right).

**Figure 2:**
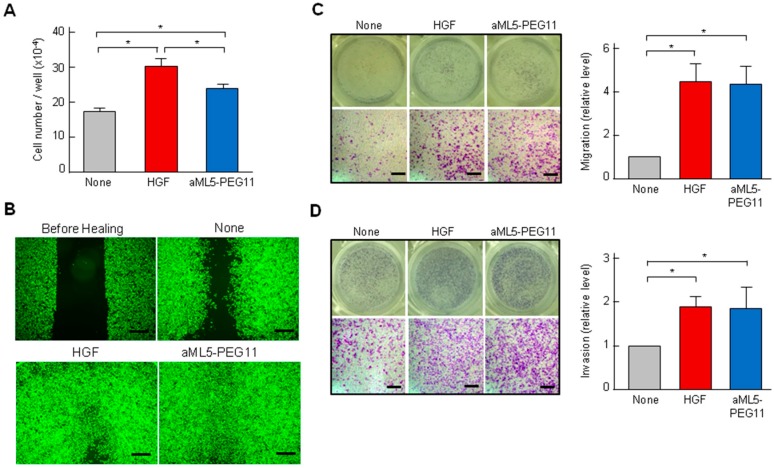
Biological responses triggered by aML5-PEG11. (**A**) Cell proliferation. HuCCT1 cells were cultured with or without 100 nM aML5-PEG11 or 3.3 nM HGF. After six days, cell numbers were counted by means of an automated cell counter. Each value indicates the mean ± SD of triplicate measurements. The asterisk indicates a significant difference (*p* < 0.05). (**B**) Scratch wound healing. After scratch wounding, HuCCT1 cells were cultured for 20 h in the absence or presence of 100 nM aML5-PEG11 or 3.3 nM HGF and images were captured. Similar results were obtained in triplicate experiments. Representative images are shown. Scale bars: 500 μm. (**C**) Cell migration. HuCCT1 cells were seeded on trans-well chambers and cultured for 20 h. Scale bar: 200 μm. (**D**) Cell invasion. HuCCT1 cells were seeded on trans-well membranes coated with type-IA collagen and cultured for 24 h. Scale bars: 200 μm. In C and D, cells that migrated through the membrane were visualized by crystal violet staining. Each value indicates the mean ± SD of triplicate measurements. The asterisk indicates a significant difference (*p* < 0.05). Representative images are shown.

We next evaluated the potential of aML5-PEG11 and HGF to induce biological responses. Since HuCCT-1 cells, which are cancer cells originated from bile duct, show biological responses both in the migration/invasion and the tubulogenesis/morphogenesis in 3-D. HuCCT1 cells were used in subsequent experiments to know the relationship between biological responses and signal transduction. Promotion of cell proliferation and migration by aML5-PEG11 and HGF were determined at the respective concentrations showing maximal MET activation. For HuCCT1 cells, 3.3 nM HGF induced cellular proliferation by 1.7-fold. aML5-PEG11 at 100 nM also facilitated cellular proliferation. However, the enhancement was lower than that by HGF (Figure 2A).

In an analysis of HuCCT1 cell migration in a scratch and healing assay, aML5-PEG11 at 100 nM was observed to enhance cell migration and recovery to a similar degree as HGF (Figure 2B). A trans-well assay was used to assess the ability to promote cell motility, migration, and invasion of cells. aML5-PEG11 (100 nM) strongly enhanced cell migration, which was comparable to HGF (Figure 2C). Furthermore, an invasion chamber assay was used to determine that aML5-PEG11 enhanced invasion of HuCCT1 cells to a degree comparable to HGF (Figure 2D). These results indicate that aML5-PEG11 is a partial agonist with a markedly reduced ability to induce MET tyrosine phosphorylation but with the ability to promote cell motility comparable to HGF.

### 2.2. Signaling Molecule Activation Profiles

**Figure 3:**
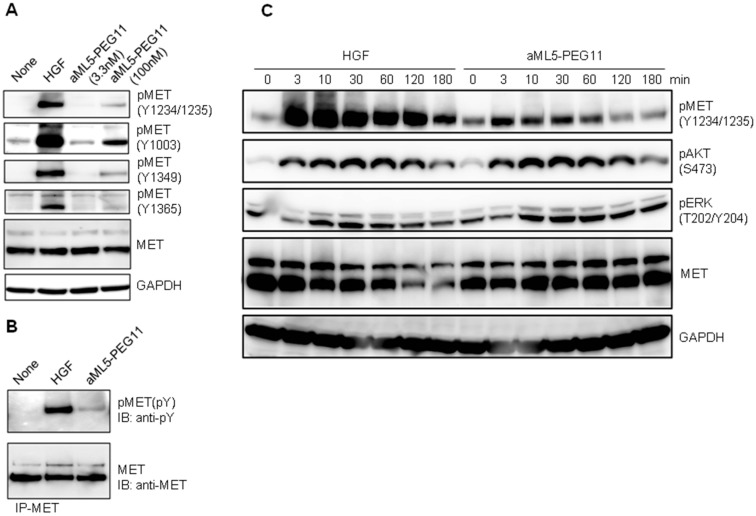
Phosphorylation profiles in the MET receptor AKT and ERK by aML5-PEG11. (**A**) Phosphorylation in individual tyrosine residues in MET receptors. EHMES-1 cells were treated with aML5-PEG11 or 3.3 nM HGF for 10 min. MET receptor phosphorylation at Y1003, Y1234/1235, Y1349, and Y1365 were measured by Western blotting. GAPDH was monitored to ensure equal loading. (**B**) MET receptor phosphotyrosine levels demonstrated by MET immunoprecipitation and Western blotting using anti-phosphotyrosine antibody. EHMES-1 cells were treated with 100 nM aML5-PEG11 or 3.3 nM HGF for 10 min. (**C**) Time-dependent changes in MET receptor, AKT, and ERK phosphorylation. HuCCT1 cells were treated with 100 nM aML5-PEG11 or 3.3 nM HGF. GAPDH was monitored to ensure equal loading. In A, B, and C, the experiments were performed once.

To investigate the mechanism permitting aML5-PEG11 to strongly induce cell migration while producing low levels of MET Y1234/1235 phosphorylation, we analyzed the characteristics of MET tyrosine phosphorylation and activation profiles of downstream signaling molecules. HGF induces autophosphorylation of several tyrosine residues within the MET receptor that are critical for biological responses. Y1003 is responsible for MET receptor endocytosis and protein stabilization [16,17], Y1234 and Y1235 are located in the kinase domain and are required for kinase activity of the receptor, and Y1349, which comprises the multi-substrate docking site, is important for recruitment of adaptor molecules and downstream signal transduction [18,19]. Additionally, phosphorylation of Y1365 mediates a morphogenic signal [20]. EHMES-1 cells were stimulated by 100 nM aML5-PEG11 or 3.3 nM HGF for 10 min and subsequently assessed for MET receptor phosphorylation at residues Y1003, Y1234/1235, Y1349, and Y1365 by Western blotting. Consistent with the cell-based ELISA results, aML5-PEG11 induced weak MET receptor phosphorylation at Y1234/1235, which was compared to that induced by HGF (Figure 3A). HGF induced robust phosphorylation at Y1003, Y1349, and Y1365 while aML5-PEG11 induced phosphorylation of Y1003, Y1349, and Y1365 to a much lower degree than HGF. MET tyrosine phosphorylation probed by the anti-phosphotyrosine antibody also confirmed the greatly reduced ability of aML5-PEG11 to induce MET tyrosine phosphorylation relative to HGF (Figure 3B).

AKT and ERK signaling pathways definitively participate in cellular responses including cell proliferation and motility. We next analyzed time-dependent changes in MET Y1234/1235 phosphorylation and phosphorylation of AKT and ERK (Figure 3C). HGF induced a strong phospho-Y1234/1235 of MET receptor within 3 min and then decreased over time, which shows a transient activation. Although aML5-PEG11 also induced phospho-Y1234/1235 of MET receptor at 3 min, the intensity was much lower than with HGF. Importantly, although 100 nM aML5-PEG11 induced marginal activation of the MET receptor, it led to AKT and ERK activation with comparable intensity and time-dependency to HGF (Figure 3C). These results suggest that the marked promotion of cell motility is induced by aML5-PEG11, which is comparable to the effects of HGF, and is attributable to the activation of AKT and ERK.

**Figure 4:**
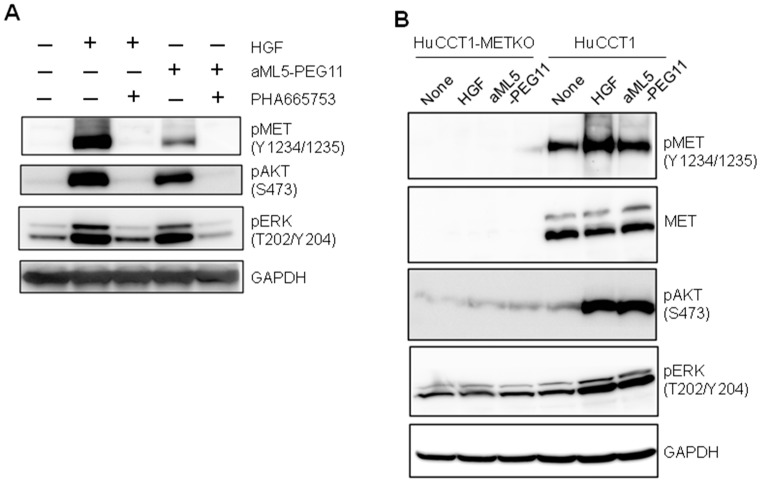
MET-receptor-dependent AKT and ERK phosphorylation by aML5-PEG11. (**A**) Effect of a selective MET tyrosine kinase inhibitor on aML5-PEG11-induced phosphorylation in MET, AKT, and ERK. EHMES-1 cells were pre-treated with or without 100 nM PHA-665752 overnight and stimulated with 100 nM aML5-PEG11 or 3.3 nM HGF for 10 min. Phospho-MET receptor at Try 1234/1235, phospho-AKT, and phospho-ERK were detected by Western blotting. GAPDH was monitored to ensure equal loading. (**B**) Phosphorylation in MET, AKT and ERK in MET receptor-knockout cells. Parental HuCCT1 cells and MET-knockout HuCCT1 cells (HuCCT1-METKO) were treated with 100 nM aML5-PEG11 or 3.3 nM HGF for 10 min. Phospho-MET receptor at Try 1234/1235, phospho-AKT, and phospho-ERK were detected by Western blotting. GAPDH was monitored to ensure equal loading. Each experiment was performed once.

To examine the possibility that aML5-PEG11 non-specifically activates other receptors, which accounts for the effect of aML5-PEG11 on cell migration, we analyzed whether AKT and ERK activation is inhibited by the selective MET tyrosine kinase inhibitor PHA-665752 (Figure 4A). PHA-665752 completely blocked MET receptor activation, p-AKT, and p-ERK induction by both aML5-PEG11 and HGF. Furthermore, we utilized MET receptor-knockout HuCCT1 cells to confirm the specificity of aML5-PEG11. HGF and aML5-PEG11 significantly induced AKT and ERK activation in HuCCT1 cells. However, they failed to activate AKT and ERK in the MET receptor-knockout HuCCT1 cells (Figure 4B). These results strongly indicate that the activation of AKT and ERK induced by aML5-PEG11 is mediated solely through the MET receptor without the involvement of other receptors or mechanisms.

### 2.3. MET Activation Profile in 3-D Epithelial Morphogenesis

**Figure 5:**
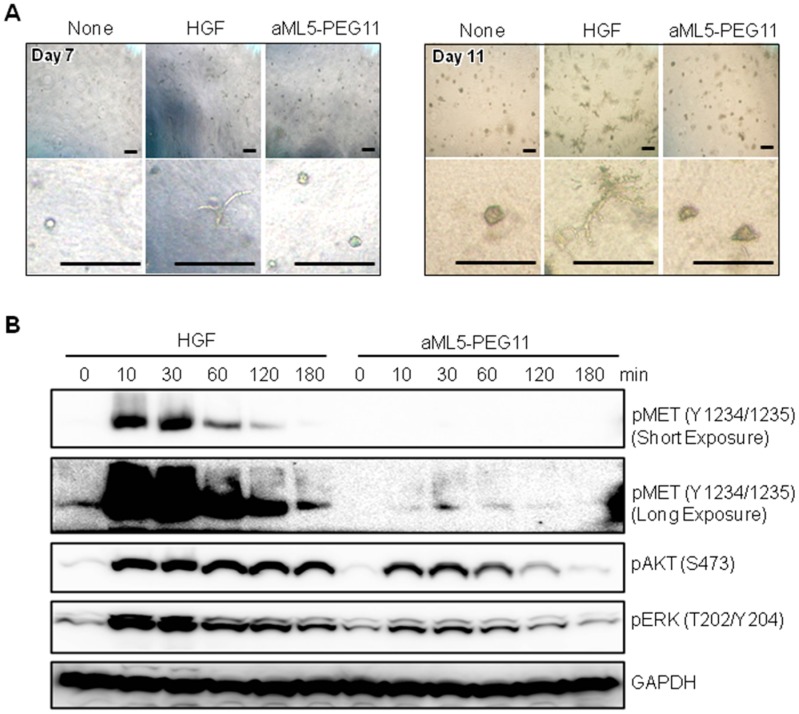
Characterization of morphogenic responses in 3-D collagen gel. (**A**) Changes in appearance of HuCCT1 cells growing in collagen gel matrix. HuCCT1 cells encapsulated in collagen gels were cultured in the absence or presence of 100 nM aML5-PEG11 or 3.3 nM HGF for 11 days. Photographs were taken on day 7 and day 11. Scale bars: 400 μm. (**B**) Time-dependent changes in MET, AKT, and ERK phosphorylation. HuCCT1 cells were untreated or treated with 100 nM aML5-PEG11 or 3.3 nM HGF. MET, AKT, and ERK phosphorylation were evaluated by Western blotting. GAPDH was monitored to ensure equal loading. Each experiment was performed once.

Since induction of dynamic morphogenesis in 3-D collagen gel is a unique biological activity mediated by the HGF-MET receptor pathway [21], we analyzed the morphogenic activity of aML5-PEG11 using this model (Figure 5A). HuCCT1 cells, which originated from the bile duct, were embedded in collagen gel and cultured for seven or 11 days in the absence or presence of 100 nM aML5-PEG11 or 3.3 nM HGF. HGF induced tubulogenesis while no morphogenic change was observed in the presence of aML5-PEG11. To clarify why aML5-PEG11 failed to induce tube formation, HuCCT1 cells were cultured in collagen gel for 24 h, treated with aML5-PEG11 or HGF for varying times, and activation of the MET receptor, AKT, and ERK was analyzed (Figure 5B). Strong MET receptor activation was induced 10 to 30 min after the addition of HGF, which was followed by a return to pre-stimulation levels. While aML5-PEG11 induced marginal MET receptor activation, the levels were much less than those induced by HGF even in the long-exposure condition. aML5-PEG11 induced AKT and ERK phosphorylation at lower levels than those induced by HGF. AKT and ERK phosphorylation induced by HGF continued for up to 180 min while AKT and ERK phosphorylation by aML5-PEG11 was greatly reduced by 120 min. This was followed by a further decrease to baseline levels at 180 min. These results suggest that, under 3-D culture conditions, aML5-PEG11 induces marginal MET receptor activation and weaker and shorter duration activation of downstream signaling pathways compared to HGF, which may be responsible for the failure to induce a morphogenic response.

**Figure 6:**
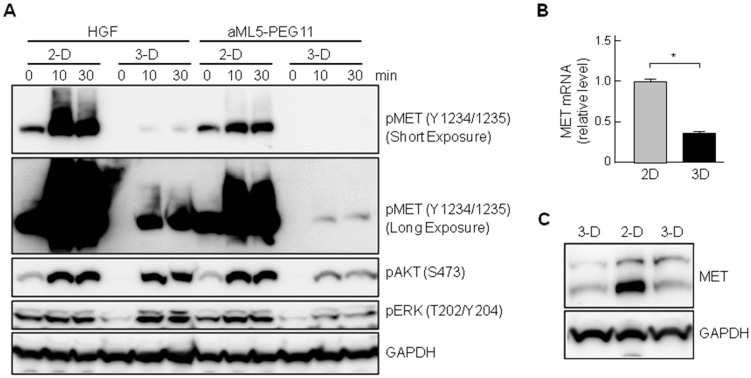
Changes in the activation and expression levels of MET receptors in 2-D and 3-D culture conditions. (**A**) Changes in MET, AKT, and ERK phosphorylation. HuCCT1 cells were cultured under 2-D (conventional monolayer on culture plate) or 3-D (in collagen gel) conditions and treated with 100 nM aML5-PEG11 or 3.3 nM HGF. Phosphorylation of the MET receptor at Try 1234/1235, AKT, and ERK was detected by Western blotting. GAPDH was monitored to ensure equal loading. (**B**) Changes in MET mRNA expression levels. HuCCT1 cells were cultured under 2-D or 3-D conditions for 24 h. MET gene expression was analyzed by quantitative RT-PCR. Each value indicates the mean ± SD of duplicate measurements. The asterisk indicates a significant difference (*p* < 0.05). (**C**) Varying MET protein levels under 2-D and 3-D culture conditions. HuCCT1 cells were cultured under 2-D or 3-D conditions for 24 h. MET receptor levels were detected by Western blotting. GAPDH was monitored to ensure equal loading.

By comparing the MET receptor phosphorylation induced by aML5-PEG11 under 2-D and 3-D culture conditions (relative to that induced by HGF under 2-D and 3-D culture conditions), we found that aML5-PEG11-induced MET receptor phosphorylation was much lower under 3-D culture conditions than under 2-D culture conditions (Figure 3 and Figure 5). To confirm our finding, MET receptor phosphorylation induced by HGF and aML5-PEG11 was analyzed in 2-D and 3-D cultures of HuCCT1 cells. Western blotting results confirmed that MET receptor activation induced by aML5-PEG11 under 3-D culture conditions was much weaker than that under 2-D culture conditions (Figure 6A). Moreover, HGF-induced MET receptor phosphorylation under 3-D culture conditions was much weaker than that under 2-D culture conditions even though the AKT and ERK activation by HGF under 2-D and 3-D culture conditions was similar (Figure 6A). The extracellular matrix influences a tissue-specific gene expression profile in a three-dimensional context, which regulates epithelial morphogenesis and function [22]. We speculated that the differences in MET receptor activation under 2-D and 3-D culture conditions might be related to differences in MET receptor expression levels at least in part. To address this possibility, HuCCT1 cells were cultured under 2-D and 3-D conditions for 24 h prior to the analysis of MET receptor expression. The MET mRNA and protein levels in the 3-D condition were 38% and 36% of those in the 2-D condition, respectively (Figure 6B,C). Thus, the decreased MET receptor expression levels in 3D may account for the decreased activation of the MET receptor and MET-mediated signaling at least in part.

### 2.4. Analysis of Morphogenesis-Related Gene Expression Profiles

**Figure 7:**
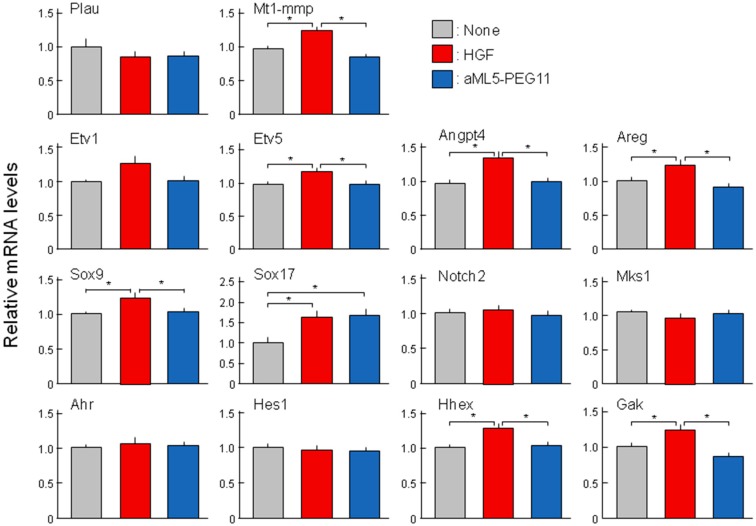
Changes in the expression of genes involved in epithelial tubulogenesis and bile duct development. HuCCT1 cells cultured in a collagen gel matrix were treated with 100 nM aML5-PEG11 or 3.3 nM HGF for 8 hours. Gene expression was analyzed by quantitative RT-PCR. The expression levels in the culture without aML5-PEG11 or HGF were regarded to be 1.0. Each value represents the mean ± SE of measurements (*n* = 7). The asterisk indicates a significant difference (*p* < 0.05).

Previous studies have examined the roles and expression of several genes involved in epithelial tubulogenesis and bile duct development including urokinase-type plasminogen activator (Plau), membrane type 1 metalloprotease (Mt1-mmp), ETS translocation variants 1 and 5 (Etv1, Etv5), angiopoietin-like 4 (Angptl4), amphiregulin (Areg), SRY-Box 9 and 17 (Sox9, Sox17), Notch2, Meckel syndrome type 1 (Mks1), aryl-hydrocarbon receptor (Ahr), hes family bHLH transcription factor 1 (Hes1), hematopoietically expressed homeobox (Hhex), and cyclin G associated kinase (Gak) [23,24,25,26,27,28,29,30]. Based on the above results that HGF but not aML5-PEG11 induced tubulogenesis in 3-D cultured HuCCT1 cells and aML5-PEG11 induced weaker and less sustained activation of AKT and ERK than HGF, we speculated that the expression of genes involved in tubulogenesis or bile duct development might be regulated by HGF while aML5-PEG11 is unlikely to regulate their expression. As expected, HGF significantly induced the expression of certain genes involved in morphogenesis including Mt1-mmp, Etv5, Angptl4, Areg, Sox9, Sox17, Hhex, and Gak. However, among these genes, only Sox17 was significantly induced by aML5-PEG11 (Figure 7). These results indicate that the inability to induce tube formation in HuCCT1 cells may be attributable to the inability of aML5-PEG11 to induce the expression of genes involved in tubulogenesis and bile duct development.

## 3. Discussion

HGF is a heterodimer composed of an α-chain and a β-chain/serine protease-like (SP) domain and the α-chain is composed of the N-terminal and four kringle domains. HGF binds to the MET receptor through two distinct interfaces: the NK1 domain in the α-chain and the β-chain [31,32,33]. There are two natural isoforms of HGF known as NK1 (the N-terminal to the first kringle domain) and NK2, which are truncated forms of HGF generated by alternative splicing [34,35,36]. NK1 facilitates cell proliferation, motility, and survival at lower efficacy than HGF [36]. In contrast, NK2 promotes cell migration but antagonized the mitogenic activity of HGF [37,38]. NK2 partially activates the MET receptor and is capable of inducing Y1234/1235 phosphorylation in the kinase domain but is incapable of inducing detectable levels of Y1349 phosphorylation in the docking site [39]. These results suggest a possibility to design or generate MET-agonists that exhibit different biological activities with different potencies compared to HGF.

In this study, we found that the partial agonist aML5-PEG11 marginally induced tyrosine phosphorylation of the MET receptor (Y1003, Y1234/1235, Y1349, Y1365) with much less efficacy than HGF. We previously found that aML5-PEG3 induced MET dimerization and tyrosine phosphorylation in a comparable strength to HGF [12]. MET dimer formation by aML5-PEG11 may be less efficient or unstable compared to aML5-PEG3 as well as HGF because of steric constraint or inappropriate linker length that are attributable to the longer linker length in aML5-PEG11 than aML5-PEG3. Nevertheless, aML5-PEG11 induced phosphorylation of ERK and AKT and strongly enhanced migration of cells to a degree comparable to HGF. On the other hand, aML5-PEG11 failed to induce tube formation under 3-D culture conditions. Failure of aML5-PEG11-induced tubulogenesis may result from the insufficient and transient activation of downstream signaling transducers such as AKT and ERK, which may further lead to deficient expression of key genes involved in epithelial tube formation and bile duct development.

aML5-PEG11 only weakly induced phosphorylation of MET tyrosine residues responsible for downstream signal transduction. However, aML5-PEG11 induced AKT and ERK activation and promoted cell migration and invasion to a degree comparable to HGF. Because non-equivalent coupling between MET tyrosine phosphorylation levels and the activation of downstream signaling and biological responses is observed with the NK2 variant [37,38,39], this appears to be a defining characteristic of MET-mediated signal transduction that may reflect a switch-like response in downstream signaling upon receptor activation [40,41,42]. When receptor activation reaches a certain threshold, a small change in ligand-dependent receptor activation can cause a large change in the activity of a downstream effector and such hypersensitivity has been observed in the regulation of cellular responses [40,41,42]. Thus, low-level activation of MET receptors in a 2-D culture may reach the threshold level for cell motility responses.

The induction of epithelial branching tubulogenesis is unique to the HGF-MET receptor pathway [21]. For HuCCT1 bile duct cancer cells in 3-D culture, aML5-PEG11 induced marginal activation/phosphorylation of the MET receptor, AKT, and ERK (5%, 30%, and 35% activities of HGF at 10 minutes after stimulation) did not influence gene expression profiles responsible for bile duct development and was unable to induce tubulogenesis. In fact, tubulogenesis is a complicated process that requires different facets of HGF signaling in a highly regulated fashion. Not only the strength but also the duration of the signaling influences the final biological outcome [43]. The inability of aML5-PEG11 to induce tubulogenesis can be explained by the greatly reduced ability of aML5-PEG11 to activate MET receptors and downstream signaling as well as the decrease of MET receptor expression in a 3-D culture.

Synthetic agonists for growth factor receptors have applications as therapeutic agents. An engineered variant of a stem cell factor with a reduced ability to dimerize Kit receptors exhibited biased activation of hematopoietic stem and progenitor cells versus mast cells and retained therapeutic efficacy without exhibiting anaphylactic off-target effects [44]. Fibroblast growth factor-19 (FGF19) exhibits metabolic effects to normalize glucose, lipid, and energy homeostasis in addition to mitogenic activity while an engineered variant FGF19 that lacks mitogenic activity was generated based on structure-activity relationships [45]. This variant FGF19 is in clinical development for the treatment of patients with non-alcoholic steatohepatitis [46]. The essential role of the HGF-MET pathway for migration and re-epithelialization during cutaneous wound healing was demonstrated in mice with keratinocyte-specific disruption of MET receptors [47] and recombinant HGF has been shown to facilitate cutaneous wound healing in animal models [48,49]. However, because proteolytic degradation of HGF impairs re-epithelialization in chronic skin ulcer patients [50] and restricted the therapeutic effects of HGF, non-native MET-agonists containing macrocyclic peptides have an advantage in treating chronic skin ulcers. Moreover, non-native and non-protein MET receptor partial agonists that potently promote epithelial cell migration with a reduced ability to promote cell proliferation represent promising therapeutic molecules with a reduced susceptibility to scar formation in the treatment of non-healing cutaneous ulcers.

Using three different MET-binding macrocyclic peptides, we generated non-protein MET ligands [12]. By using appropriate PEG linkers, MET-agonists with bivalent macrocyclic peptides promoted cell proliferation, migration, and tubulogenesis with comparable efficacy to native HGF. In this study, we demonstrated that aML5-PEG11 induces partial MET activation. This weak activation confers the characteristics of a partial MET-agonist with biological activities that differ from native HGF in an extracellular context-dependent manner. Our study suggests the possibility of generating unique partial MET-agonists by changing linker length and/or the combination of MET-binding macrocyclic peptides.

## 4. Materials and Methods

### 4.1. Cells, HGF, Peptide, and Reagents

The EHMES-1 human mesothelioma cell line was kindly provided by Dr. Hamada (Ehime University, Japan) and cultured in RPMI1640 medium supplemented with 10% fetal bovine serum (FBS) (Sigma-Aldrich, St. Louis, MO, USA). The HuCCT1 human bile duct carcinoma cell line was obtained from the Japanese Cancer Research Resources Bank and cultured in RPMI1640 medium supplemented with 10% FBS. Recombinant human HGF prepared from the conditioned medium of CHO cells stably expressing human HGF was provided from Kringle Pharma Inc. (Osaka, Japan). aML5-PEG11 (Figure 1A) was synthesized, as described previously [12]. Anti-pMET (Y1234/1235), -pMET (Y1003), -pMET (Y1349), -MET, -pERK (T202/Y204), -pAKT (S473), -pTyr-HRP, and -GAPDH antibodies were purchased from Cell Signaling Technology Japan (Tokyo, Japan). The anti-pMET (Y1365) antibody was purchased from SIGMA (St. Louis, MO, USA).

### 4.2. Establishment of MET Deficient Cells by Genome Editing

Platinum TALEN plasmids were constructed by using the Platinum Gate TALEN Kit (Addgene) as described previously [51] with some modifications. In brief, each DNA-binding module was assembled into modified ptCMV-136/63-VR vectors containing the CAG promoter by using the two-step Golden Gate cloning method. The target sequence was 5′-TGTTTACCTTGGTGCAGaggagcaatggg gagTGTAAAGAGGCACTAGCA-3′ where uppercase and lowercase letters indicate TALEN target sequences and the spacer sequence, respectively.

The 1.0-kb fragments of the MET gene used for the 5′ and 3′ arms of the targeting vector were amplified by PCR from HuCCT1 cells genomic DNA using the following primer sets: 5′-TTAAATACGTGCTAGTGAGAGGTTTATCTGCCAAA-3′ and 5′-CCAGCGGCCCGCTAGGTCATACCTATTTAGTTTATATT-3′ (for the 5′ arm); 5′-CGCGCCTTAAGTCGAATAGGGAATGCACACATGG-3′ and 5′-TGGGAAGCTTGTCGAAAGTCCGAGATGAATGTGAA-3′ (for the 3′ arm). These fragments were then sub-cloned into the NheI site (for the 5′ arm) and SalI site (for the 3′ arm) of the DT-A-pA/loxP/PGK-Puro-pA/loxP vector [52] to create pKO-hMet-Puro, which was co-transfected with the TALEN plasmids into HuCCT1 cells. After puromycin selection, surviving clones were screened to detect homologous recombination by genomic PCR using the following primer pair: 5′-ATCCAGGGTTAGTCTTTGGC-3′ and 5′-TTGTACTCAGCAACCTTCTG-3′. MET deficiency was further confirmed through the immunoprecipitation followed by Western blotting using the anti-MET antibody.

### 4.3. Cell-Based Phospho-MET Receptor Assay

Cells were seeded in 96-well black micro-clear plates (Greiner Bio-One) at a density of 8 × 10^3^ cells per well for EHMES-1 cells or 1.5 × 10^4^ cells per well for HuCCT1 cells and cultured for 24 h. The cells were stimulated with aML5-PEG11 or HGF in RPMI1640 medium supplemented with 10% FBS for 10 min, washed once with ice-cold PBS, and fixed with 4% paraformaldehyde in PBS for 30 min at room temperature. After washing three times with PBS, the cells were blocked with 5% goat serum, 0.02% Triton X-100 in PBS for 30 min at room temperature, and incubated with anti-phospho-MET (Y1234/1235) (D26) XP rabbit mAb (1:1000 diluted in PBS with 1% goat serum) at 4 °C overnight. The cells were washed three times with PBS and incubated in an HRP-conjugated anti-rabbit goat antibody (1:1000 diluted in PBS with 1% goat serum) for 1 h. After washing four times with PBS, the tyrosine-phosphorylated MET receptor was detected by an ImmunoStar LD reagent (Wako, Japan) and measured by using the ARVO MX (Perkin Elmer, Norwalk, CT, USA). Relative MET receptor phosphorylation levels were calculated as follows: (sample chemiluminescence unit—mock control chemiluminescence unit)/(highest chemiluminescence unit—mock control chemiluminescence unit).

### 4.4. Cell Growth Assay

The cells were seeded at 5 × 10^3^ cells per well in 24-well plates. After 24 h, cells were treated with HGF or aML5-PEG11 in RPMI1640 medium supplemented with 5% FBS and culture medium was refreshed every 3 days. After 6 days, the cells were harvested with a trypsin–EDTA solution, suspended in the culture medium, and the cell number was determined by using a Countess Automated Cell Counter (Life Technologies, Carlsbad, CA, USA).

### 4.5. Scratch Wound-Healing, Migration, and Invasion Assays

The scratch wound-healing assay was conducted by seeding cells (5 × 10^4^) in 96-well plates and culturing for 24 h. The confluent monolayer of cells was wounded with a 1000-μL pipette tip and washed with culture medium. The cells were then cultured in the absence or presence of HGF or aML5-PEG11 for 20 h. The cells were subsequently stained with 5 μg/mL calcein-AM (Dojindo) at 37 °C for 15 min and images were acquired by using BIOREVO BZ-9000 (Keyence, Osaka, Japan) technology.

The migration and invasion assays were conducted by using trans-well chambers (6.5 mm diameter trans-well with 8 μm pores, Corning). HuCCT1 (2 × 10^4^) cells cultured in 100 μL RPMI1640 medium supplemented with 0.5% FBS and were plated into the upper insert while 600 μL RPMI1640 medium supplemented with 0.5% FBS with or without aML5-PEG11 or HGF was added to the bottom chamber. The cells were cultured for 20 h and fixed with 4% paraformaldehyde in PBS. Cells attached to the bottom of the insert were stained with 0.2% crystal violet in 20% methanol. To prepare the invasion assay, the inserts were pre-coated with 200 μg/mL Cellmatrix type IA collagen solution (Nitta Gelatin, Osaka, Japan). The cells were plated and cultured as above and the cells attached to the bottom of the insert were stained with 0.2% crystal violet in 20% methanol.

### 4.6. Branching Morphogenesis Assay

HuCCT1 (1 × 10^4^) cells were suspended in 500 μL Cellmatrix type IA collagen solution (Nitta Gelatin, Osaka, Japan) in culture medium in a 24-well plate and allowed to gelate for 30 min at 37 °C, according to the manufacturer’s instructions. After gelation, 500 μL RPMI1640 supplemented with 10% FBS with or without HGF or aML5-PEG11 was added to each well. Culture medium was refreshed every three days.

### 4.7. Western Blotting

The cells were seeded at 6 × 10^5^ cells per well in six-well plates and serum starved for 4 h. The cells were stimulated with HGF or aML5-PEG11 for the desired time. After washing with ice-cold PBS, the cells were lysed in 150 μL 1 × SDS-PAGE Laemmli sample buffer with ultrasonication (Vibra-cell). For the 3-D culture, 8 × 10^5^ HuCCT1 cells were suspended in 100 μL Cellmatrix type IA collagen solution (Nitta Gelatin, Osaka, Japan) and seeded in 48-well plates. After gelation, 500 μL RPMI1640 medium supplemented with 0.5% FBS was added to each well and cultured for 24 h. The cells were stimulated with HGF or aML5-PEG11 for the desired time. The cells were lysed in 50 μL 4 × SDS-PAGE Laemmli sample buffer with ultrasonication. The cell lysates were subjected to SDS-PAGE and electroblotted onto PVDF membranes. The membranes were treated with a primary antibody (diluted at 1:1000) and HRP-conjugated secondary antibodies (Dako, Carpinteria, CA, USA) (diluted at 1:2000). Chemiluminescence was visualized and quantitated by using an ImmunoStar LD (Wako, Japan).

### 4.8. Quantitative Real-Time PCR

The cells were seeded at 1 × 10^5^ per well in a 300 μL Cellmatrix type IA collagen solution (Nitta Gelatin, Osaka, Japan) in a 24-well plate and allowed to gelate for 60 min at 37 °C, according to the manufacturer’s instruction. The cells were stimulated with HGF or aML5-PEG11 for 8 h. Total RNA was prepared by using Seposal-RNA I Super G (Nacalai Tesque, Kyoto, Japan) and 1 µg of total RNA was reverse transcribed into cDNA by using a reverse transcriptase kit (SuperScript VILO, Invitrogen, Carlsbad, CA, USA), according to the manufacturer’s protocol. Quantitative PCR was performed on a 7500 real time PCR system (Applied Biosystems, Foster City, CA, USA) by using the FastStart Universal SYBR Green Master (Roche, Mannheim, Germany). The primers are listed in Table 1.

### 4.9. Statistical Analysis

The data were analyzed by a one-way ANOVA followed by the Tukey’s multiple comparisons test using GraphPad Prism 7. Values of *p* < 0.05 were considered statistically significant.

## Figures and Tables

**Table 1:** Primers used for q-PCR.

mRNAs	Sequences
**Mt1-mmp**	F: 5′-CACTGCCTACGAGAGGAAGG-3′
	R: 5′-TTGGGGTACTCGCTATCCAC-3′
**Angptl4**	F: 5′-GTCCACCGACCTCCCGTTA-3′
	R: 5′-CCTCATGGTCTAGGTGCTTGT-3′
**Areg**	F: 5′-GAGCCGACTATGACTACTCAGA-3′
	R: 5′-TCACTTTCCGTCTTGTTTTGGG-3′
**Etv1**	F: 5′-TGGCAGTTTTTGGTAGCTCTTC-3′
	R: 5′-CGGAGTGAACGGCTAAGTTTATC-3′
**Etv5**	F: 5′-CAGTCAACTTCAAGAGGCTTGG-3′
	R: 5′-TGCTCATGGCTACAAGACGAC-3′
**Sox9**	F: 5′-AGCGAACGCACATCAAGAC-3′
	R: 5′-CTGTAGGCGATCTGTTGGGG-3′
**Sox17**	F: 5′-GTGGACCGCACGGAATTTG-3′
	R: 5′-GGAGATTCACACCGGAGTCA-3′
**Notch2**	F: 5′-CCTTCCACTGTGAGTGTCTGA-3′
	R: 5′-AGGTAGCATCATTCTGGCAGG-3′
**Hhex**	F: 5′-TCAGAATCGACGCGCTAAATG-3′
	R: 5′-AGAGCTATCCAAAGAAGCACCT-3′
**Gak**	F: 5′-CCACCCGAACATTGTCCAGTT-3′
	R: 5′-AGAACCGTGTCGCACGAAA-3′
**Mks1**	F: 5′-GCAAGAAAAACCGACGAATCTTT-3′
	R: 5′-TCGCTCGACCAAGAATGAAGG-3′
**Ahr**	F: 5′-ACATCACCTACGCCAGTCGC-3′
	R: 5′-TCTATGCCGCTTGGAAGGAT-3′
**Hes1**	F: 5′-ACGTGCGAGGGCGTTAATAC-3′
	R: 5′-ATTGATCTGGGTCATGCAGTTG-3′
**Met**	F: 5′-AGATGAATGTGAATATGAAGTATC-3′
	R: 5′-CAGTCTTGTACTCAGCAACCT-3′
**Gapdh**	F: 5′-GAGTCAACGGATTTGGTCGT-3′
	R: 5′-GACAAGCTTCCCGTTCTCAG-3′

## Abbreviations

HGF	hepatocyte growth factor
RTKs	receptor tyrosine kinases
EpoR	erythropoietin receptor
EGFR	epidermal growth factor receptor
RaPID	random nonstandard peptides integrated discovery
ERK	extracellular signal-regulated kinase
AKT	protein kinase B
GAPDH	glyceraldehyde-3-phosphate dehydrogenase
Plau	plasminogen activator urokinase
Mt1-mmp	Membrane type 1 metalloprotease
Etv	ETS translocation variant
Angptl4	Angiopoietin Like 4
Areg	Amphiregulin
Sox	SRY-Box
Mks1	Meckel syndrome type 1
Ahr	aryl-hydrocarbon receptor
Hes1	hes family bHLH transcription factor 1
Hhex	hematopoietically expressed homeobox
Gak	cyclin G associated kinase

## References

[1] Levin D., Schneider W.M., Hoffmann H.H., Yarden G., Busetto A.G., Manor O., Sharma N., Rice C.M., Schreiber G. (2014). Multifaceted activities of type I interferon are revealed by a receptor antagonist. Sci. Signal..

[2] Macdonald-Obermann J.L., Pike L.J. (2014). Different epidermal growth factor (EGF) receptor ligands show distinct kinetics and biased or partial agonism for homodimer and heterodimer formation. J. Biol. Chem..

[3] Moraga I., Richter D., Wilmes S., Winkelmann H., Jude K., Thomas C., Suhoski M.M., Engleman E.G., Piehler J., Garcia K.C. (2015). Instructive roles for cytokine-receptor binding parameters in determining signaling and functional potency. Sci. Signal..

[4] Moraga I., Wernig G., Wilmes S., Gryshkova V., Richter C.P., Hong W.J., Sinha R., Guo F., Fabionar H., Wehrman T.S. (2015). Tuning cytokine receptor signaling by re-orienting dimer geometry with surrogate ligands. Cell.

[5] Riese D.J. (2011). Ligand-based receptor tyrosine kinase partial agonists: New paradigm for cancer drug discovery?. Expert Opin. Drug Discov..

[6] Yea K., Xie J., Zhang H., Zhang W., Lerner R.A. (2015). Selection of multiple agonist antibodies from intracellular combinatorial libraries reveals that cellular receptors are functionally pleiotropic. Curr. Opin. Chem. Biol..

[7] Freed D.M., Bessman N.J., Kiyatkin A., Salazar-Cavazos E., Byrne P.O., Moore J.O., Valley C.C., Ferguson K.M., Leahy D.J., Lidke D.S. (2017). EGFR ligands differentially stabilize receptor dimers to specify signaling kinetics. Cell.

[8] Kimura T., Kaburaki H., Miyamoto S., Katayama J., Watanabe Y. (1997). Discovery of a novel thrombopoietin mimic agonist peptide. J. Biochem..

[9] Tarasova A., Haylock D.N., Meagher L., Be C.L., White J., Nilsson S.K., Andrade J., Cartledge K., Winkler D.A. (2013). Potent agonists of a hematopoietic stem cell cytokine receptor, c-Mpl. ChemMedChem.

[10] Simonneau C., Leclercq B., Mougel A., Adriaenssens E., Paquet C., Raibaut L., Ollivier N., Drobecq H., Marcoux J., Cianférani S. (2015). Semi-synthesis of a HGF/SF kringle one (K1) domain scaffold generates a potent in vivo MET receptor agonist. Chem. Sci..

[11] Ueki R., Ueki A., Kanda N., Sando S. (2016). Oligonucleotide-based mimetics of hepatocyte growth factor. Angew. Chem. Int. Ed. Engl..

[12] Ito K., Sakai K., Suzuki Y., Ozawa N., Hatta T., Natsume T., Matsumoto K., Suga H. (2015). Artificial human Met agonists based on macrocycle scaffolds. Nat. Commun..

[13] Furlan A., Kherrouche Z., Montagne R., Copin M.C., Tulasne D. (2014). Thirty years of research on met receptor to move a biomarker from bench to bedside. Cancer Res..

[14] Petrini I. (2015). Biology of MET: A double life between normal tissue repair and tumor progression. Ann. Transl. Med..

[15] Imamura R., Matsumoto K. (2017). Hepatocyte growth factor in physiology and infectious diseases. Cytokine.

[16] Abella J.V., Peschard P., Naujokas M.A., Lin T., Saucier C., Urbé S., Park M. (2005). Met/Hepatocyte growth factor receptor ubiquitination suppresses transformation and is required for Hrs phosphorylation. Mol. Cell Biol..

[17] Kong-Beltran M., Seshagiri S., Zha J., Zhu W., Bhawe K., Mendoza N., Holcomb T., Pujara K., Stinson J., Fu L. (2006). Somatic mutations lead to an oncogenic deletion of met in lung cancer. Cancer Res..

[18] Ma P.C., Maulik G., Christensen J., Salgia R. (2003). c-Met: Structure, functions and potential for therapeutic inhibition. Cancer Metastasis Rev..

[19] Ponzetto C., Bardelli A., Zhen Z., Maina F., dalla Zonca P., Giordano S., Graziani A., Panayotou G., Comoglio P.M. (1994). A multifunctional docking site mediates signaling and transformation by the hepatocyte growth factor/scatter factor receptor family. Cell.

[20] Weidner K.M., Sachs M., Riethmacher D., Birchmeier W. (1995). Mutation of juxtamembrane tyrosine residue 1001 suppresses loss-of-function mutations of the met receptor in epithelial cells. Proc. Natl. Acad. Sci. USA.

[21] Montesano R., Matsumoto K., Nakamura T., Orci L. (1991). Identification of a fibroblast-derived epithelial morphogen as hepatocyte growth factor. Cell.

[22] Bissell M.J., Barcellos-Hoff M.H. (1987). The influence of extracellular matrix on gene expression: Is structure the message?. J. Cell Sci. Suppl..

[23] Delannoy-Courdent A., Fauquette W., Dong-Le Bourhis X.F., Boilly B., Vandenbunder B., Desbiens X. (1996). Expression of c-ets-1 and uPA genes is associated with mammary epithelial cell tubulogenesis or neoplastic scattering. Int. J. Dev. Biol..

[24] Weaver S.A., Wolters B., Ito N., Woskowicz A.M., Kaneko K., Shitomi Y., Seiki M., Itoh Y. (2014). Basal localization of MT1-MMP is essential for epithelial cell morphogenesis in 3D collagen matrix. J. Cell Sci..

[25] Chotteau-Lelievre A., Montesano R., Soriano J., Soulie P., Desbiens X., de Launoit Y. (2003). PEA3 transcription factors are expressed in tissues undergoing branching morphogenesis and promote formation of duct-like structures by mammary epithelial cells in vitro. Dev. Biol..

[26] Ciarloni L., Mallepell S., Brisken C. (2007). Amphiregulin is an essential mediator of estrogen receptor alpha function in mammary gland development. Proc. Natl. Acad. Sci. USA.

[27] Raynaud P., Carpentier R., Antoniou A., Lemaigre F.P. (2011). Biliary differentiation and bile duct morphogenesis in development and disease. Int. J. Biochem. Cell Biol..

[28] Zong Y., Stanger B.Z. (2011). Molecular mechanisms of bile duct development. Int. J. Biochem. Cell Biol..

[29] Harstad E.B., Guite C.A., Thomae T.L., Bradfield C.A. (2006). Liver deformation in Ahr-null mice: Evidence for aberrant hepatic perfusion in early development. Mol. Pharmacol..

[30] Lee D.W., Zhao X., Yim Y.I., Eisenberg E., Greene L.E. (2008). Essential role of cyclin-G-associated kinase (Auxilin-2) in developing and mature mice. Mol. Biol. Cell.

[31] Matsumoto K., Kataoka H., Date K., Nakamura T. (1998). Cooperative interaction between α- and β-chains of hepatocyte growth factor on c-Met receptor confers ligand-induced receptor tyrosine phosphorylation and multiple biological responses. J. Biol. Chem..

[32] Kirchhofer D., Yao X., Peek M., Eigenbrot C., Lipari M.T., Billeci K.L., Maun H.R., Moran P., Santell L., Wiesmann C. (2004). Structural and functional basis of the serine protease-like hepatocyte growth factor beta-chain in Met binding and signaling. J. Biol. Chem..

[33] Stamos J., Lazarus R.A., Yao X., Kirchhofer D., Wiesmann C. (2004). Crystal structure of the HGF β-chain in complex with the Sema domain of the Met receptor. EMBO J..

[34] Miyazawa K., Kitamura A., Naka D., Kitamura N. (1991). An alternatively processed mRNA generated from human hepatocyte growth factor gene. Eur. J. Biochem..

[35] Chan A.M., Rubin J.S., Bottaro D.P., Hirschfield D.W., Chedid M., Aaronson S.A. (1991). Identification of a competitive HGF antagonist encoded by an alternative transcript. Science.

[36] Cioce V., Csaky K.G., Chan A.M., Bottaro D.P., Taylor W.G., Jensen R., Aaronson S.A., Rubin J.S. (1996). Hepatocyte growth factor (HGF)/NK1 is a naturally occurring HGF/scatter factor variant with partial agonist/antagonist activity. J. Biol. Chem..

[37] Day R.M., Cioce V., Breckenridge D., Castagnino P., Bottaro D.P. (1999). Differential signaling by alternative HGF isoforms through c-Met: Activation of both MAP kinase and PI 3-kinase pathways is insufficient for mitogenesis. Oncogene.

[38] Tolbert W.D., Daugherty-Holtrop J., Gherardi E., Vande Woude G., Xu H.E. (2010). Structural basis for agonism and antagonism of hepatocyte growth factor. Proc. Natl. Acad. Sci. USA.

[39] Mungunsukh O., Lee Y.H., Bottaro D.P., Day R.M. (2016). The hepatocyte growth factor isoform NK2 activates motogenesis and survival but not proliferation due to lack of Akt activation. Cell Signal..

[40] Heinrich R., Neel B.G., Rapoport T.A. (2002). Mathematical models of protein kinase signal transduction. Mol. Cell.

[41] Ferrell J.E., Machleder E.M. (1998). The biochemical basis of an all-or-none cell fate switch in Xenopus oocytes. Science.

[42] Azeloglu E.U., Iyengar R. (2015). Signaling networks: Information flow, computation, and decision making. Cold Spring Harb. Perspect. Biol..

[43] Rosário M., Birchmeier W. (2003). How to make tubes: Signaling by the Met receptor tyrosine kinase. Trends Cell Biol..

[44] Ho C.C.M., Chhabra A., Starkl P., Schnorr P.J., Wilmes S., Moraga I., Kwon H.S., Gaudenzio N., Sibilano R., Wehrman T.S. (2017). Decoupling the functional pleiotropy of stem cell factor by tuning c-Kit signaling. Cell.

[45] Wu X., Ge H., Lemon B., Vonderfecht S., Baribault H., Weiszmann J., Gupte J., Gardner J., Lindberg R., Wang Z. (2010). Separating mitogenic and metabolic activities of fibroblast growth factor 19 (FGF19). Proc. Natl. Acad. Sci. USA.

[46] Harrison S.A., Rinella M.E., Abdelmalek M.F., Trotter J.F., Paredes A.H., Arnold H.L., Kugelmas M., Bashir M.R., Jaros M.J., Ling L. (2018). NGM282 for treatment of non-alcoholic steatohepatitis: A multicentre, randomised, double-blind, placebo-controlled, phase 2 trial. Lancet.

[47] Chmielowiec J., Borowiak M., Morkel M., Stradal T., Munz B., Werner S., Wehland J., Birchmeier C., Birchmeier W. (2007). c-Met is essential for wound healing in the skin. J. Cell Biol..

[48] Bevan D., Gherardi E., Fan T.P., Edwards D., Warn R. (2004). Diverse and potent activities of HGF/SF in skin wound repair. J. Pathol..

[49] Yoshida S., Matsumoto K., Tomioka D., Bessho K., Itami S., Yoshikawa K., Nakamura T. (2004). Recombinant hepatocyte growth factor accelerates cutaneous wound healing in a diabetic mouse model. Growth Factors.

[50] Buchstein N., Hoffmann D., Smola H., Lang S., Paulsson M., Niemann C., Krieg T., Eming S.A. (2009). Alternative proteolytic processing of hepatocyte growth factor during wound repair. Am. J. Pathol..

[51] Sakuma T., Ochiai H., Kaneko T., Mashimo T., Tokumasu D., Sakane Y., Suzuki K., Miyamoto T., Sakamoto N., Matsuura S. (2013). Repeating pattern of non-RVD variations in DNA-binding modules enhances TALEN activity. Sci. Rep..

[52] Ninagawa S., Okada T., Sumitomo Y., Kamiya Y., Kato K., Horimoto S., Ishikawa T., Takeda S., Sakuma T., Yamamoto T. (2014). EDEM2 initiates mammalian glycoprotein ERAD by catalyzing the first mannose trimming step. J. Cell Biol..

